# Efficient Removal of Mercury from Wastewater Solutions by a Nitrogen-Doped Hyper-Crosslinked Polyamine

**DOI:** 10.3390/polym16172495

**Published:** 2024-08-31

**Authors:** Khalid Al Ghamdi, Aqeel Ahmad, Gheorghe Falca, Meshal Nawaf Alrefaeia, Othman Charles S. Al-Hamouz

**Affiliations:** 1 Department of Chemistry, King Fahd University of Petroleum and Minerals (KFUPM), Dhahran 31261, Saudi Arabia; g200614340@kfupm.edu.sa (K.A.G.); s202265900@kfupm.edu.sa (M.N.A.); 2 Interdisciplinary Research Center for Refining and Advanced Chemicals, King Fahd University of Petroleum and Minerals (KFUPM), Dhahran 31261, Saudi Arabia; aqeel.ahmad@kfupm.edu.sa; 3 Interdisciplinary Research Center for Membranes and Water Security, King Fahd University of Petroleum and Minerals (KFUPM), Dhahran 31261, Saudi Arabia; gheorghe.falca@kfupm.edu.sa

**Keywords:** polyamine, polycondensation, mercury removal, environmental remediation, adsorption

## Abstract

Mercury, a highly toxic metal and pollutant, poses a significant risk to human health and the environment. This study describes the synthesis of a new nitrogen-doped heteroaromatic hyper-crosslinked polyamine (*HCPA*) via the polycondensation of 2,6-diaminopyrazine and tris(4-formylphenyl)amine for the efficient removal of mercury ions from aqueous solutions. The *HCPA* polymer was characterized by solid-state ^13^C-NMR and FT-IR spectroscopy. A powder X-ray diffraction and thermogravimetric analysis showed that the polymer was semicrystalline in nature and stable up to 500 °C. Adsorption isotherms indicated that mercury adsorption occurred via mono- and multilayer adsorption by *HCPA*, as depicted by the Langmuir, Freundlich, and Redlich–Peterson isotherm models, with a maximum adsorption capacity of *q*_m_ = 333.3 mg/g. Adsorption kinetic models suggested that the adsorption process was fast and effective, reaching equilibrium within 20 min. Thermodynamics of the adsorption process revealed that it was endothermic and spontaneous in nature due to the positive Δ*H*^0^ of 48 kJ/mol and negative Δ*G*^0^ values of −4.5 kJ/mol at 293 K. Wastewater treatment revealed 98% removal of mercury indicating the selective nature of *HCPA*. Finally, *HCPA* exhibited excellent performance and regeneration up to three cycles, demonstrating its great potential as an adsorbent for environmental remediation applications.

## 1. Introduction

An important worldwide problem is the contamination of water in aquatic systems by harmful metals [1]. Mercury, the second most deadly heavy metal after plutonium, is particularly harmful to human life and aquatic environments [2]. Studies have identified elemental and inorganic mercury as prevalent in crude oil and gas condensate [3]. Inorganic mercury, existing in mercury (I) and mercury (II) oxidation states [4,5], is predominantly water-soluble [6]. Consequently, it threatens subterranean aquifers, freshwater, marine, and soil ecosystems [2]. The pollution of these critical resources undermines natural equilibrium and harms people and animals. When harmful metals are released into the environment, they impede biological growth, which in turn harms plants and animals and eventually affects the general stability of ecosystems [7].

Recent research has underscored that exposure to Hg(II) ions poses significant risks to the reproductive health, central nervous system, kidneys, as well as sensory and psychological functions [1]. Its robust mobility in environmental matrices, substantial bioaccumulation in food webs, and acute toxicity to organisms necessitate stringent regulatory measures. The World Health Organization (WHO) and the US Environmental Protection Agency (EPA) have set maximum allowable Hg levels in wastewater and drinking water at 10 μg/L and 2 μg/L, respectively [8,9]. Around two-thirds of the Hg(II) ions found in nature come from human activity, namely from sectors such as the electrical, paint, pharmaceutical, oil refining, gold mining, and battery production industries [7,10]. Therefore, reducing the concentration of mercury is a crucial step to mitigate the potential threat posed by Hg to living organisms [10]. 

Several approaches, including filtration, electrolysis, solvent extraction, adsorption, distillation, crystallization, and reverse osmosis, have been devised and used to reduce the harmful impacts of mercury (II) ions on the environment [2]. However, among these methods, adsorption stands out as the most preferred option due to its accessibility to a diverse range of adsorbents, demonstrating promising efficiency, cost-effectiveness, environmental compatibility, ease of application, scalability, and energy efficiency [11]. However, the efficacy of adsorption is contingent upon the selection of an appropriate adsorbent. This highlights the continuous need for research into the identification and development of efficient adsorbents. 

Over the past decade, a variety of materials, encompassing aerogels [12], zeolite [13], metal nanoparticles [14] like activated carbon, graphene [15], and naturally occurring materials [16], have been scrutinized to determine their effectiveness in removing Hg(II) ions from water solutions [7]. However, the use of adsorption-based technology using traditional adsorbents is limited by poor adsorption capacity and slow adsorption kinetics [1,17]. Only a restricted subset of these substances has undergone evaluation for their adsorption efficacy in multicomponent solutions, a scenario frequently encountered in industrial wastewater treatment processes. Effective heavy metal removal from aqueous solutions is mostly dependent on the design of effective adsorbents, with the presence of chelating functionalities being a key determinant of performance enhancement [2,18]. Chelating resins containing nitrogen or sulfur-based ligands demonstrate exceptional selectivity for divalent transition metal cations, such as Hg (II), because of the high attraction between these atoms and the targeted metal ions. 

Polymer sorbents have garnered considerable attention for their superior adsorption capacity, efficiency, and ease of preparation compared to other materials [1]. These polymers are engineered to incorporate functional groups that have a high affinity for mercury, including oxygen in aromatic foldarands, aerogels and nitrogen in polyamines, and sulfur in polythiols [19,20]. Despite advancements, the development of new polymer sorbents that can quickly, selectively, and effectively remove Hg ions from water is still a major difficulty in both industry and environmental remediation [7]. Research indicates that adsorption materials containing polyamine functional groups, characterized by multiple nitrogen atoms within their molecular structures, hold promise for their robust adsorption properties and selectivity towards Hg (II) ions owing to the strong binding affinity between the metal ions and nitrogen [20]. The synthesis of novel porous polymers from easily accessible molecular monomers without the need for catalysts presents a significant challenge in polymer chemistry [10]. Despite its potential as a high-affinity adsorbent for mercury ions, the utilization of pyrazine as a monomeric unit remains underexplored in the literature [2,7]. By leveraging the rich NH_2_ and OH groups inherent in pyrazine, engineered functional adsorbents with enhanced adsorption capabilities for mercury ions were developed through molecular structure modification, demonstrating improved adsorption affinities and active adsorption site densities [10,21].

This study presents the design and synthesis of a novel polymer for the efficient removal of mercury from wastewater solutions with the ability to regenerate and reuse for potential application as an adsorbent for heavy metal remediation.

## 2. Materials and Methods

### 2.1. Materials

2,6-Diaminopyrazine (99%) and tris(4-formylphenyl)amine (99%) were purchased from Oakwood Chemicals (Estill, SC, USA). The 99.8% anhydrous N,N-Dimethyl formamide (99.8%) and mercury (II) chloride salt were bought from Sigma Aldrich (Steinheim, Germany). A 1000 mg/L standard stock solution was diluted with deionized (DI) water to prepare the Hg (II) solution. All chemicals were used in their original form.

### 2.2. Methods

#### 2.2.1. Synthesis of HCPA

**Scheme 1:**
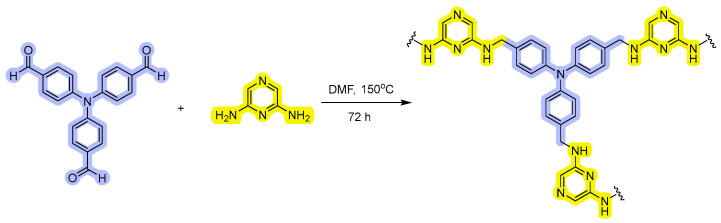
Synthesis of *HCPA* for the removal of mercury.

The *HCPA* polymer was synthesized via a polycondensation reaction. In a typical reaction, tris(4-formylphenyl) amine (TFPA) (0.005 mol, 1.6432 g) and 2,6-Diaminopyrazine (DAP) (0.015 mol, 1.6577 g) were dissolved in 25 mL DMF of in a 75 mL heavy-walled pressure vessel. The mixture was stirred until a homogeneous solution was achieved. The solution mixture was heated to 150 °C under stirring for 72 h. After the completion of the reaction, the mixture was cooled to the ambient temperature, and the resulting solid product was separated by filtration and then washed with DMF and with deionized water three times to ensure the complete removal of solvent or unreacted chemicals. Finally, the polymer was subjected to a drying process in a vacuum oven at a temperature of 60 °C for 24 h until it reached a consistent weight (yield: 2.75 g, 83.2%) (Scheme 1).

#### 2.2.2. Equipment

The structural features possessed by *HCPA* polymer were assessed through solid-state ^13^C NMR analysis using a Bruker Ultrashield 400WB (Billerica, MA, USA) plus instrument operating at 400 Hz. The structure was evaluated by FTIR, with spectra taken on a Nicolet iS50 FT-IR (Mountain, WI, USA) spectrometer across 32 scans at a resolution of 4 cm^−1^. To investigate the thermal stability of *HCPA*, a Linseis STA PT 1600 (Robbinsville, NJ, USA) instrument was used for TGA analysis; the polymer sample was heated at a rate of 10 °C/min from 20 to 700 °C under Air. The crystallinity of the material was examined via XRD analysis performed on the polymer powder using an Ultima IV X-ray (Tokyo, Japan) diffractometer equipped with a CuKα radiation source operating at 40 kV and 40 mV (PXRD). SEM analysis was conducted by a FEI Quattro Scanning Electron Microscope, Seoul, Republic of Korea. Assessment of the polymer’s capability to adsorb Hg ions was carried out using a mercury analyzer (Model MA-3000, Nippon Instruments North America, College Station, TX, USA). Wastewater analysis was conducted using an inductively coupled plasma mass spectrometer (Thermo Scientific Element 2, Waltham, MA, USA).

#### 2.2.3. Adsorption Experiments

Mercury adsorption tests were carried out as follows. Firstly, mercury chloride salt was dissolved in DI water at a specific concentration and pH that was adjusted by the addition of 0.1 M sodium hydroxide or 0.1 M nitric acid. The effect of pH was conducted to determine the effect of pH by combining 10 mg of *HCPA* polymer with 10 mL of a 20 ppm Hg solution at a specific pH range (1–6) at 25 °C and stirring for 24 h. In order to examine the impact of initial concentration on adsorption capacity, 10 mg of *HCPA* polymer was added to 10 mL of mercury solution containing a range of 20 ppm to 715 ppm at a pH of 5.5 over the course of 4 h. To test how the adsorption capacity changed with time, a 20-ppm mercury solution was mixed with 10 mg of *HCPA* polymer in a pH 5.5 solution. The effect of temperature on the adsorption capacity of *HCPA* was systematically explored across a temperature gradient of 20, 35, and 50 °C. After finishing the adsorption experiment the polymer was filtered out and the concentration of Hg in the filtrate solution was measured, the adsorption capacity and % removal of the polymer were calculated using the following equations [10,22]:

(1) $$q_{e}=\frac{\left(C_{f}-C_{e}\right)\times V}{m}$$

(2) $$\%\;Removal=\frac{C_{f}-C_{e}}{C_{f}}\times 100\%$$

where *q*_e_ is equilibrium adsorption capacity (mg/g), *C*_f_ indicates the mercury concentrations of the feed, and *C*_e_ represents the mercury concentration at equilibrium measured in g/L. *V* represents the volume in L and *m* is the mass of the polymer in g.

#### 2.2.4. Reuse and Regeneration of HCPA Polymer for the Adsorption of Hg (II) Ions

The reusability of *HCPA* polymer was performed as follows: 20 mg of *HCPA* polymer was immersed in 20 mL of Hg solution at pH 5.5 for 24 h. The supernatant of the solution was separated from the polymer via centrifugation at 7800 rpm for 5 min, and the adsorbed mercury was released by adding a solution of HNO_3_ (0.1 M). The polymer was immersed in the acid solution for about 2 h. The polymer pH was adjusted by repetitive washing with DI water and the procedure was repeated for a total of three cycles [23]. 

## 3. Results

A novel polymer was synthesized via the polycondensation reaction of tris(4-formylphenyl) amine (TFPA) and 2,6-Diaminopyrazine (DAP). The polycondensation reaction yielded a polymer with -CH_2_-NH- linkage between the monomeric units to provide a strong covalent three-dimensional structure with strong adsorption sites filled with amine groups to be capable of adsorbing heavy metals such as mercury ions [24,25,26]. This type of polycondensation reaction produces important types of polymers that are cheap to synthesize due to the absence of catalysts and high-reactivity functional groups, which are easy to design based on their end use and have good thermal and chemical stability due to their strong covalent bonds [27,28].

### 3.1. Structural Performance of HCPA Polymer

**Figure 1:**
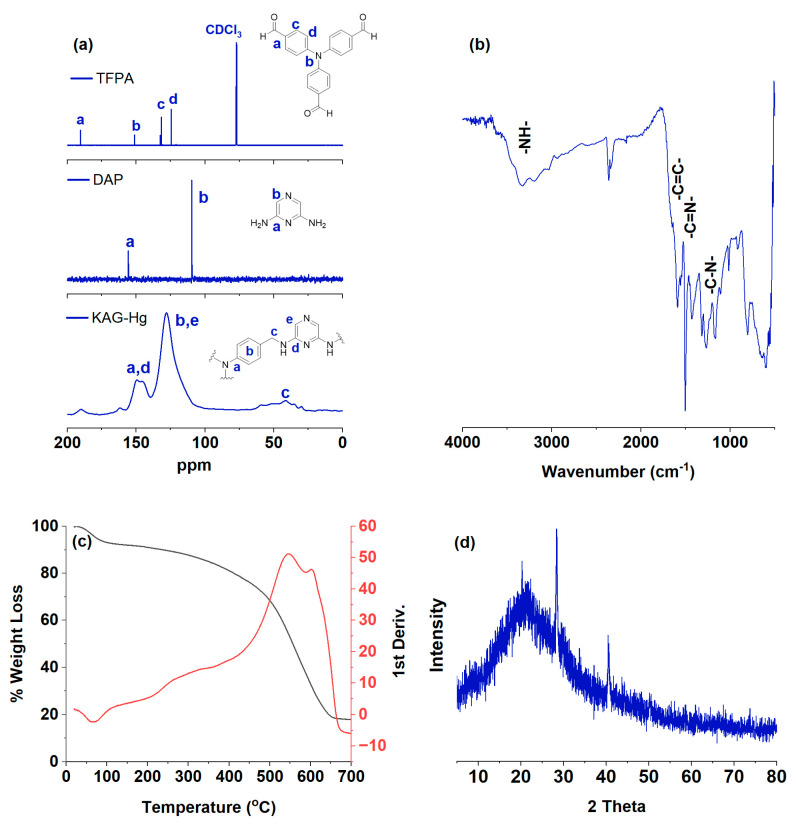
Structural properties of the *HCPA* polymer: (**a**) Solid ^13^C-NMR; (**b**) FT-IR spectra; (**c**) TGA; (**d**) PXRD pattern.

NMR and FT-IR are essential analytical tools in the structural elucidation of the polymer, providing complementary information on molecular structure and functional groups, that are vital for optimizing material properties and applications. Figure 1a provides the ^13^C-NMR of the synthesized *HCPA* polymer. From Figure 1a, it can be noticed that the aliphatic (-CH_2_-) between the two monomeric units appeared at 40 ppm [7,29]. The peaks observed at 155.3 and 129.4 ppm correspond to pyrazine and benzene aromatic (-C=C-). The peaks observed at around 190 ppm correspond to the unreacted formaldehyde (-C(O)-H groups at the terminals of the polymer chains. A peak at 163.7 ppm can be ascribed to imine (-C=N-) linkage that can form from the reaction of the amine with formaldehyde groups [30]. After the NMR study, the synthesized polymer was analyzed by FT-IR. The FT-IR spectrum shown in Figure 1b reveals an absorption band at 3400 cm^−1^ that corresponds to the -N-H stretching frequency [7]. Two distinct adsorption peaks observed at approximately 1600 cm^−1^ and 1400 cm^−1^ can be attributed to the stretching vibrations of -C=N- and -C–N- bonds, respectively, indicating specific imine and pyrazine groups within the polymer. The thermal properties of the polymer were evaluated by TGA, as shown in Figure 1c. From the Figure 1c, it can be noticed that the initial weight decrease appeared before 100 °C, which can be attributed to the evaporation of solvents trapped within the cross-linked polymers. After that, the polymer showed a second small degradation at ~270 °C due to the decomposition of the unreacted terminal formaldehyde groups at the end of the polymer chains. The third and major decomposition of the polymer chains began at 520 °C, indicating the thermal stability of the polymer structure [31]. These results align with previous findings [32] concerning porous materials, indicating that the robust cross-linkages present within the structures contribute to enhanced thermal resistance of the polymers. The PXRD and SEM results of the synthesized polymers are shown in Figure 1d and Figure S1 (Supporting Information), indicating that HCPA is semi-crystalline in nature and the SEM results confirm that the crystals are embedded in the agglomerates of amorphous chains of the polymer [7].

### 3.2. Effect of pH

**Figure 2:**
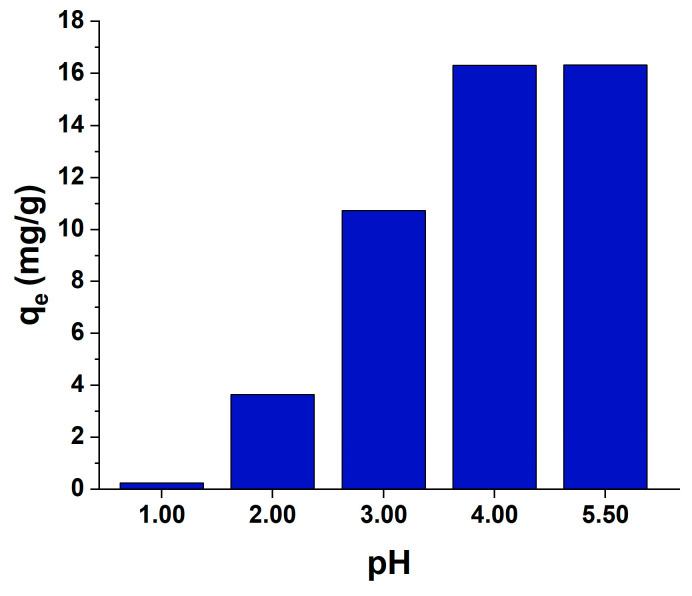
The effect of pH on the adsorption capacity of *HCPA* polymer.

Figure 2 illustrates the impact of pH on the adsorption capacity of *HCPA* toward mercury (II) ions ranging from pH 1 to 5.5; pH values above 5.5 chemical precipitation of Hg (II) ions will affect the study. The results reveal that the adsorption capacity increases with the increase of pH, indicating the competitive adsorption between H^+^ and Hg (II) ions. The quantity of Hg (II) ions adsorbed exhibited an escalating trend with rising pH levels, reaching its max at pH ≥ 4.0. Given its pH-sensitive nature, the protonation of functional groups naturally transpires in solutions with low pH and the formation of positive sites (-NH^+^-, -NH^2+^-) on the adsorbent surface [33]. Diminishing the concentration of H^+^ ions in solutions with pH > 3 augmented the adsorption capacity to >16 mg/g. Furthermore, this behavior may be ascribed to the strong coordination between the amine’s nitrogen atom lone pairs, which actively couple with mercury ions to improve mercury adsorption. 

### 3.3. Effect of Initial Concentration on the Adsorption Capacity of HCPA Polymer

The evaluate the adsorption behavior of *HCPA* polymer toward Hg (II) ions, the effect of the initial concentration of Hg (II) ions on the adsorption capacity was investigated. These investigative procedures comprise a series of static adsorption assays conducted at diverse concentrations at the interface of liquid and solid phases, thereby facilitating the derivation of an adsorption isotherm. Subsequently, this isotherm underwent mathematical modeling for further elucidation. For this objective, the equilibrium adsorption capacity (*q*_e_) of *HCPA* with mercury (II) was determined by treating the polymer with various aqueous concentrations of Hg (II) ions ranging from 20 to 715 ppm. To discern the equilibrium adsorption behavior, three distinct models were employed, namely Langmuir, Freundlich, and Redlich–Peterson.

**Figure 3:**
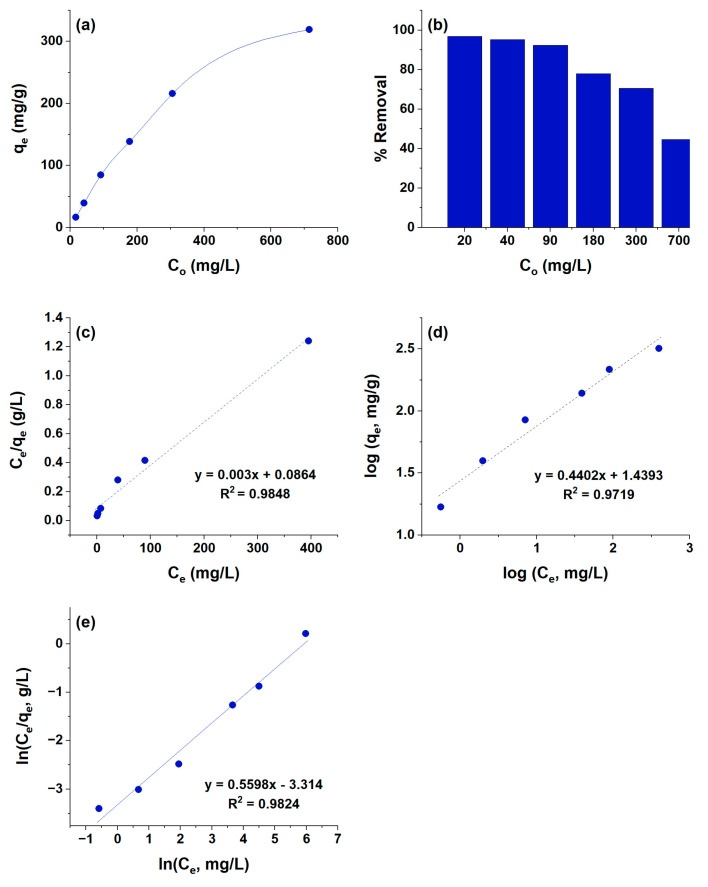
(**a**) Initial concentration effect on the adsorption capacity of the *HCPA* polymer; (**b**) removal % of Hg (II) ions by the *HCPA* polymer; (**c**) Langmuir isotherm model; (**d**) Freundlich isotherm model; (**e**) Redlich–Peterson isotherm model.

Figure 3a illustrates the trend of *HCPA* polymer adsorption capacity with increasing concentrations of Hg (II) ions. The Figure 3 shows that the adsorption capacity improved when the Hg (II) ion concentration rose, demonstrating the polymer’s improved performance as a result of its functionality. The % removal decreased with increasing the concentration, which is explained by the polymer reaching maximum adsorption. To better investigate the adsorption behavior of *HCPA* polymer, the adsorption experimental data were subjected to the Langmuir model. This implies that the adsorption process involves the formation of a single layer of adsorbate molecules with consistent adsorption energy (Figure 3c). The following equations are representations of the linear and non-linear forms of the Langmuir isotherm model [34]:

(3) $$q_{e}=\frac{K_{l}q_{m}C_{e}}{1+K_{l}C_{e}}$$

(4) $$\frac{C_{e}}{q_{e}}=\frac{C_{e}}{q_{m}}+\frac{1}{K_{l}q_{m}}$$

where *q*_m_ and *q*_e_ (mg/g) represent the maximum binding and equilibrium adsorption capacities, respectively. *C*_e_ is the concentration at equilibrium (mg/L) and *K*_l_ is the Langmuir constant (L/mg) [35]. The Langmuir isotherm model constants are shown in Table 1. With a correlation value of 0.9848 and a maximal capacity for adsorption (*q*_m_) of 333.3 mg/g, the experimental data support the model well. The high adsorption capacity is attributed to the high concentration of nitrogen atoms, which creates a high density of active sites in the *HCPA* polymer for the adsorption of Hg (II) ions.

On the other hand, the Freundlich isotherm model instead accounts for a multilayer heterogeneous adsorption process [36]. The non-linear and linear forms of the isotherm model are depicted as follows:

(5) $$q_{e}=K_{f}\;C_{e}^{1/n}$$

(6) $$\log\left(q_{e}\right)=\frac{1}{n}\log\left(c_{e}\right)+\log\left(K_{f}\right)$$

The Freundlich model constants, represented by *K*_f_ and 1/*n*, are employed to characterize adsorption behavior. As shown in Figure 3d and Table 1, the experimental data also fit well with the isotherm model with *R*^2^ of 0.9719, with a 1/*n* value of 0.4402, which lies in the favorable adsorption range of the model of 0 < 1/*n* < 1 [37].

**Figure 4:**
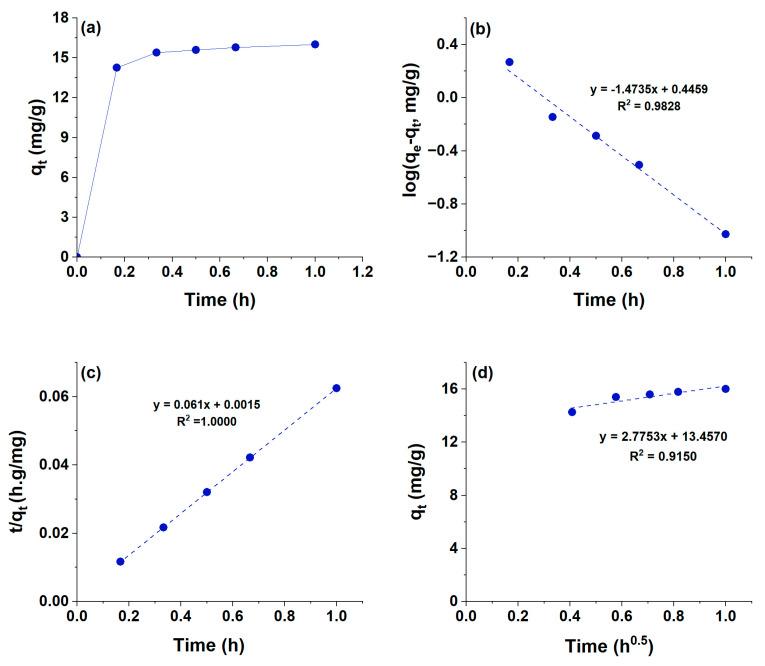
Adsorption kinetics: (**a**) effect of time on the adsorption capacity of *HCPA*, (**b**) pseudo-first order, (**c**) pseudo-second order, (**d**) intraparticle diffusion.

Since both the Langmuir and Freundlich models accurately describe the experimental data, we applied the Redlich–Peterson model, which is utilized to assess the homogeneity/heterogeneity of the adsorption process, as it encompasses the features of both the Freundlich and Langmuir isotherm models [34]. As depicted in Figure 4 (Table 1), the non-linear and linear forms of the model can be expressed as follows:

(7) $$q_{e}=\frac{K_{r}C_{e}}{1+\alpha C_{e}^{\beta }}$$

(8) $$ln\left(\frac{C_{e}}{q_{e}}\right)=\beta \;ln\left(C_{e}\right)-ln\left(k_{r}\right)$$

where *β* is an exponent that ranges between 0 and 1, *α* is a constant and is expressed in L/mg, and *K*_r_ represents the Redlich–Peterson constant (L/g).

Analysis of the of the regression value of the model (*R*^2^ = 0.9824) suggests that the adsorption process behavior of *HCPA* polymer exhibits a hybrid nature, characterized by a combination of mono- and multi-layer adsorption mechanisms, potentially showing disparate adsorption energies across various adsorption sites. From the *β* value in the model (*β* = 0.5598), the model suggests that the adsorption follows mono- and multilayer adsorption with the value being closer to 1 assuming that the adsorption process is controlled more by the monolayer homogenous adsorption [38]. This could be explained by the different types of nitrogen atoms embedded in the polymer structure: two nitrogen atoms are present, the pyridinic nitrogen and the secondary amine. Two mechanisms of binding can be assumed. First, at pH 5.5, the speciation form of mercury is Hg(OH)_2_, which will have two modes of contact with the polymer. First, the polymer pyridinic nitrogens will bind with Hg^2^⁺ directly, which could lead to a monolayer adsorption mechanism. Second, secondary amines can bind in two ways: directly from nitrogen to Hg^2^⁺ and through H-bonding with the OH group in the Hg(OH)_2_ species, leading to multilayer adsorption, as predicted by the Redlich–Peterson isotherm model [39].

### 3.4. Effect of Time on the Adsorption Capacity of HCPA Polymer

The analysis of mercury adsorption over time allowed us to determine the affinity between the *HCPA* polymer and Hg (II) ions. For elucidating the nature and mechanism of adsorption, the experimental data were processed using intraparticle diffusion kinetic models, pseudo-second-order models, and pseudo-first-order models. According to the graphical presentation in Figure 4a, the adsorption of Hg (II) ions with *HCPA* polymer was rapid and required 30 min to reach equilibrium. Therefore, the *HCPA* polymer shows high potential as an adsorbent for the removal of Hg (II) ions from aqueous systems. The pseudo-first-order kinetic model was first applied to the experimental data; this model suggests that the adsorption mechanism is physisorption and is proportional to the unoccupied active site equilibrium. The fitting of the experimental data to the linear equation of the model is shown in Equation (9), and the fitted experimental results are presented in Figure 1b, where *q*_e_ (mg/g) and *q*_t_ (mg/g) denote adsorption capacities at equilibrium and at a selected time *t*, respectively. *k*_1_ (*h*^−1^) is the constant of the model [40].

(9) $$\log\left(q_{e}-q_{t}\right)=\log q_{e}-\frac{k_{l}}{2.203}t$$

The example of the pseudo-second-order model is shown in Figure 4c, and its application is described by Equations (10) and (11). The kinetic constant of the pseudo-second-order, *k*_2_ (g/mg·h), and the initial adsorption rate, *H* (mg/g·h), are detailed below, respectively.

(10) $$\frac{t}{q_{t}}=\frac{1}{k_{2}\;q_{e}^{2}}+\frac{t}{q_{e}}$$

(11) $$H=k_{2}\;q_{e}^{2}$$

The intraparticle diffusion model can be expressed by Equation (12) (Weber–Morris), where *C* is the constant of the model and *k*_p_ represents the diffusion rate.

(12) $$q_{t}=k_{p}\;t^{1/2}+C$$

where *k*_p_ represents the rate constant of intraparticle diffusion (mg/g·h) and *C* represents the boundary layer thickness. 

As reported in Table 2, the calculated regression values (*R*^2^) values of the fitted data were 0.9828 and 1.00 for the pseudo-first-order and pseudo-second-order models, respectively. Hence, from these results, it can be concluded that the pseudo-second-order model fits the adsorption experimental data. This suggests that the adsorption rate is controlled by chemisorption, which involves electronic migration between the polymer and mercury [37]. 

The intraparticle diffusion model is applied to elucidate the adsorption mechanism, particularly to identify which step forms the bottleneck in the entire process. The adsorption kinetic model is composed of three critical phases: initially, during boundary diffusion, the adsorbate spreads across the external surface of the adsorbent. This is followed by intraparticle diffusion, where the adsorbate permeates the pores of the adsorbent. The final phase involves the adsorbate occupying the inner sites of the adsorbent [37,41,42,43]. According to Figure 4d, the experimental data did not pass through the origin, indicating that the adsorption process is not solely controlled by intraparticle diffusion, but involve two stages, namely diffusion of Hg(II) ions onto the pores of the polymer followed by intraparticle diffusion [44].

### 3.5. Effect of Temperature on the Adsorption Capacity of HCPA Polymer

**Figure 5:**
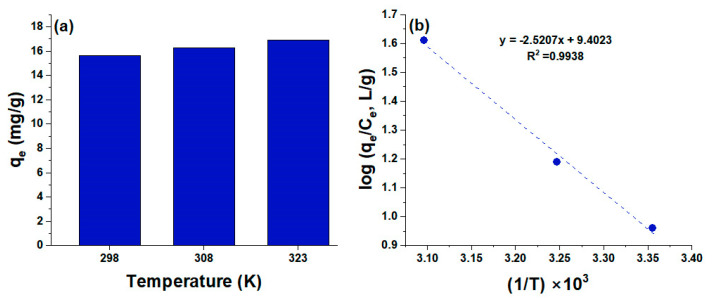
(**a**) The effect of temperature on the adsorption capacity of the *HCPA* polymer; (**b**) the Van’t-Hoff thermodynamic model.

The impact of temperature variations on the adsorption capabilities of *HCPA* polymer was systematically explored across a temperature gradient of 20, 35, and 50 °C, as shown in Figure 5a. The adsorption capacity exhibited an increase in the adsorption capacity with increasing temperature, reaching an adsorption capacity of *q*_e_ = 17 mg/g at 50 °C. The increase in the adsorption capacity could be attributed to the expansion of the polymer at higher temperatures allowing more Hg (II) ions to diffuse in the polymer and adsorbed. To better understand the thermodynamic process Gibbs free energy, enthalpy, and entropy of the adsorption process were calculated using the following equations [37,45,46]:

(13) $$\Delta G^{{}^{\circ}}=\Delta H^{{}^{\circ}}-T\Delta S^{{}^{\circ}}$$

(14) $$\log\left(\frac{q_{e}}{C_{e}}\right)=\frac{\Delta S^{{}^{\circ}}}{2.303R}-\frac{\Delta H^{{}^{\circ}}}{2.303RT}$$

where ∆*G*° denotes the Gibbs free energy (kJ/mol), ∆*H*° stands for the system’s enthalpy (kJ/mol), *T* represents the temperature (K), and ∆*S*° signifies the system’s entropy (J/mol).

According to the data in Table 3 and illustrated in Figure 5b utilizing the Van’t-Hoff thermodynamic model, the results revealed that the adsorption is endothermic in nature. The Gibbs free energy values were negative, assuming that the adsorption process is spontaneous, and became more favorable at higher temperatures [37]. Therefore, an increase in temperature would bestow a thermodynamic advantage upon the polymer-adsorbent system, resulting in an enhanced final adsorption capacity. 

### 3.6. Reuse and Regeneration of HCPA Polymer

**Figure 6:**
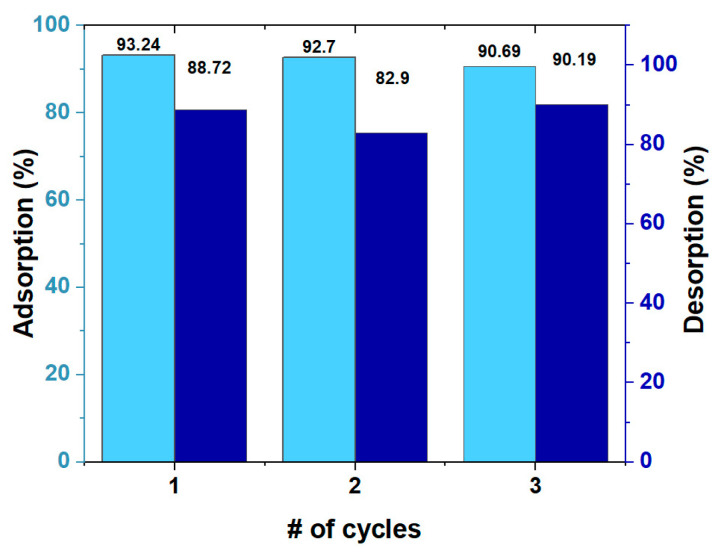
Mercury adsorption/desorption cycles.

The overall pH-scaled performance in the comprehensive affinity study demonstrates that the polymer can be used for selective binding and releasing mercury (II) ions at different pH levels. Furthermore, this property is utilized in the desorption and regeneration experiments to support the possibility of its commercial use, as reported in Figure 6. The pH during the adsorption phase was a constant 5.5, while in the second step, the pH of the aqueous solution was 1. As shown in Figure 6, the percentage of mercury (II) ions absorbed by the *HCPA* during the adsorption and desorption processes remained similar, varying from 93.24 to 90.69% in the case of the adsorption, whereas the desorption ranged from 90.19 to 88.72%. The observations imply that the polymer is effective even at a low pH, and the adsorbent’s functionality is maintained for the first three cycles. 

### 3.7. Wastewater Treatment

To acquire knowledge about the capabilities of *HCPA* for the selective removal of Hg(II) ions from a real wastewater sample, a real wastewater sample spiked with 20 mg/L of Hg (II) ions was tested, and the results are shown in Table 4.

The results shown in Table 4 show the selective nature of *HCPA* polymer in the removal of Hg (II) ions from a real wastewater sample. The efficient removal of Hg (II) ions could be attributed to the high concentration of nitrogen atoms present in the *HCPA* polymer, which acts as a strong Lewis base toward the empty orbitals of Hg (II) ions facilitating the selective nature of *HCPA* to remove ~98% of Hg (II) ions in wastewater. The comparative analysis of the adsorption capacity of Hg (II) ions among different adsorbent materials is shown in Table 5. 

## 4. Conclusions

This study introduces a novel nitrogen-doped hyper-crosslinked polyamine (HCPA) material, demonstrating significant scientific value and practical applicability. The HCPA’s exceptional thermal stability, semicrystalline morphology, and abundant nitrogen functional groups contribute to its outstanding performance as a mercury adsorbent. Adsorption studies revealed a high affinity for mercury ions, attributed to the synergistic effect of monolayer and multilayer adsorption mechanisms. The pseudo-second-order kinetics and endothermic nature of the adsorption process further highlight the HCPA’s potential for efficient mercury removal. Moreover, the material’s excellent reusability and selective removal of mercury from real wastewater samples underscore its practical applicability in environmental remediation. While HCPA exhibits promising characteristics, future studies could explore its performance under varying conditions, such as in the presence of competing ions or at different pH levels. Additionally, investigating the potential for scaling up the synthesis process and optimizing the adsorption conditions would further enhance the HCPA’s commercial viability. In conclusion, the HCPA represents a significant advancement in the field of mercury adsorption. Its unique combination of properties offers a promising solution for addressing mercury contamination in aqueous environments, contributing to sustainable water management and environmental protection.

## Figures and Tables

**Table 1:** Langmuir, Freundlich, and Redlich–Peterson isotherm model parameters for the adsorption of Hg (II) ions by the *HCPA* polymer.

	Langmuir Isotherm Model		
*HCPA*	*q*_m_ (mg/g)	*K*_l_ (L/mg)	*R* ^2^
	333.3	0.0347	0.9848
	Freundlich Isotherm Model		
	*K*_f_ (mg^(1−1/*n*)^L^1/*n*^g^−1^)	*1/n*	*R* ^2^
	27.50	0.4402	0.9719
	Redlich–Peterson Isotherm Model		
	*β*	*K*_r_ (L/g)	*R* ^2^
	0.5598	27.4948	0.9824

**Table 2:** Kinetic parameters for pseudo-first-order and pseudo-second-order models for mercury (II) adsorption onto *HCPA*.

**Pseudo-First-Order Model**			
*q*_e(exp)_ (mg/g)	*q*_e(calc.)_ (mg/g)	*k*_1_ (min^−1^)	*R* ^2^
16.6	2.791	3.246	0.9828
**Pseudo-Second-Order Model**			
*q*_e(exp)_ (mg/g)	*q*_e(calc.)_ (mg/g)	*k*_2_ (min^−1^)	*R* ^2^
16.6	16.39	2.481	1.0000
**Intraparticle Diffusion**			
	*k*_p_ (mg/g.h)	*C*	*R^2^*
	2.7753	13.4570	0.9150

**Table 3:** Thermodynamic parameters for the adsorption of Hg (II) ions by the *HCPA* polymer.

Polymer	$\Delta H^{0}\;\left(kJ/mol\right)$	$\Delta S^{0}\;\left(J/mol\right)$	$\Delta G^{0}\;\left(kJ/mol\right)$	T (K)
*HCPA*	48.26	180.0	−4.511	293.15
			−7.211	308.15
			−9.912	323.15

**Table 4:** The concentration of metal ions of a wastewater sample spiked with 20 (mg/L) Hg (II) ions before and after adsorption by the *HCPA* polymer.

pH: 8.124			
Metal Ion	Before Adsorption (mg/L)	After Adsorption (mg/L)	Removal %
Hg	22.34	0.484	97.8
Fe	32.10	32.10	0.0
B	5.10	3.0	41.2
Mg	195	182.4	6.5
Ca	422.4	270	36.1
Ag	2.40	2.40	0.0
Cr	2.10	0.60	71.4
Pb	3.00	2.10	30.0
K	121.2	121.2	0.0
Cu	1.80	0.30	83.3
Zn	8.10	2.70	66.7

**Table 5:** The Hg (II) ion adsorption capability of several adsorbent materials.

Adsorbent	*q*_m_ (mg/g)	Reference
*HCPA*	333.30	This study
SBA-15-SH	195.60	[47]
γ-AlOOH	114.00	[48]
MXR-1	0.41	[7]
Novel polydithiocarbamate	22.03	[2]
Coal-based activated carbon	48.00	[49]
MWCNTs-Fe_3_O_4_	239.00	[50]
CAS15-S	86.00	[51]
Quartz	16.52	[52]

## Data Availability

The original contributions presented in the study are included in the article/Supplementary Material, and further inquiries can be directed to the corresponding author.

## Supplementary Materials

The following supporting information can be downloaded at: https://www.mdpi.com/article/10.3390/polym16172495/s1. Figure S1. SEM image of KAG-Hg.
