# Supplementary material for: Efficient Removal of Mercury from Wastewater Solutions by a Nitrogen-Doped Hyper-Crosslinked Polyamine
Source: Polymers (Basel). 2024 Aug 31;16(17):2495. doi: 10.3390/polym16172495 (PMC11398116; doi:10.3390/polym16172495)
Supplement: Supplementary file 1 [file polymers-16-02495-s001.zip › polymers-3172252-supplementary.pdf]

Supplementary Information

Efficient Removal of Mercury from Wastewater Solutions by a Nitrogen-Doped Hyper-Crosslinked Polyamine

Khalid Al Ghamdi [1], Aqeel Ahmad [2], Gheorghe Falca [3], Meshal Nawaf Alrefaeia [1] and Othman Charles S. Al-Hamouz [1,2,*]

[1] Department of Chemistry, King Fahd University of Petroleum and Minerals (KFUPM), Dhahran 31261, Saudi Arabia
[2] Interdisciplinary Research Center for Refining and Advanced Chemicals, King Fahd University of Petroleum and Minerals, Dhahran 31261, Saudi Arabia
[3] Interdisciplinary Research Center for Membranes and Water Security, King Fahd University of Petroleum and Minerals, Dhahran 31261, Saudi Arabia
* Correspondence: othmanc@kfupm.edu.sa

S1. SEM

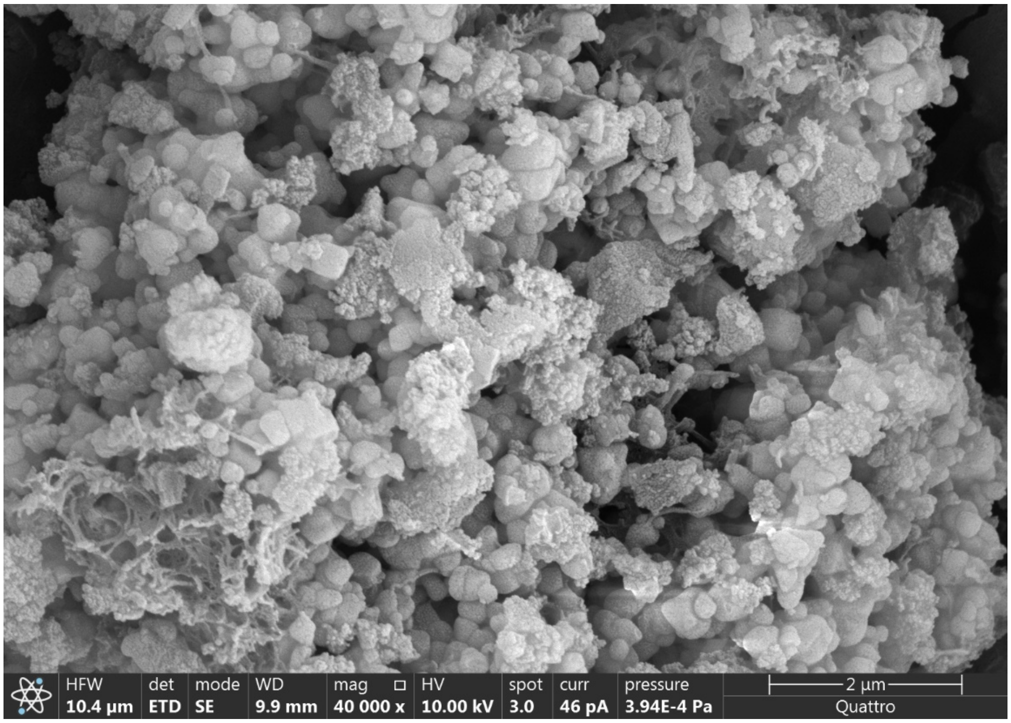

Figure S1. SEM image of KAG-Hg
